# Streptococcus agalactiae as a Primary Cause of Infective Endocarditis With Septic Emboli in an Undiagnosed Rheumatic Mitral Stenosis Patient: An Encounter in a US-Based Safety-Net Hospital

**DOI:** 10.7759/cureus.37802

**Published:** 2023-04-18

**Authors:** Dhan B Shrestha, Jurgen Shtembari, Leanne Sowunmi, Aarya Adhikari, Tilak Joshi

**Affiliations:** 1 Department of Internal Medicine, Mount Sinai Hospital, Chicago, USA; 2 Department of Internal Medicine, Ross University School of Medicine, Bridgetown, BRB; 3 Department of Internal Medicine, Chitwan Medical College, Bharatpur, NPL

**Keywords:** rheumatic heart disease, splenic infarction, infective endocarditis, streptococcus agalactiae, septicemia

## Abstract

Splenic infarct is a rare sequel of *Streptococcus agalactiae* infective endocarditis (IE). We report a case of a 43-year-old woman with multiple comorbidities diagnosed with a splenic infarct secondary to group B *Streptococcus* IE. The development of a splenic hematoma complicated the hospital course. This case highlights the less common etiology of IE and the potential complications.

## Introduction

*Streptococcus agalactiae* is a normal component of the gastrointestinal (GI) and genitourinary (GU) flora [1]. While it is the most common cause of postpartum infections and neonatal sepsis, it is a rare cause of infective endocarditis (IE) [2]. IE due to *S. agalactiae* is still associated with a poor prognosis, significant mortality and morbidity, and a high rate of embolic events, despite improvements in medical and surgical treatment [3]. Group B *Streptococcus* (GBS) IE incidence has increased from 8.1 cases per 100,000 in 2008 to 10.9 cases per 100,000 in 2016, with a mortality of 6.5-38% among nonpregnant adults [3]. *S. agalactiae* infections are thought to develop in people with persistent underlying conditions, such as cancer, diabetes, liver cirrhosis, and human immunodeficiency virus (HIV) infection [4]. Complicated cases of IE due to GBS can cause the seeding of cardiac emboli in various organs [3]. Early presentation, massive vegetations, notably the mitral valve, big arterial emboli, and a high death rate were all characteristics of these illnesses [5].

Before antibiotics, emboli were a significant cause of death (70-78%), but these days, they only account for 15-35% of deaths [5]. The spleen (44%), kidneys (56%), coronary arteries (40-60%), and cerebral arteries (40-60%) are the most common locations of emboli [5]. Splenic infarct is a rare sequel of GBS IE [3].

Here, we present a case of endocarditis in a woman with an extensive past medical history, diagnosed with GBS IE with septic emboli.

## Case presentation

We present the case of a 43-year-old female who presented to the emergency department with altered mental status, somnolence, fevers, tachycardia, and severe hyperglycemia. She had been previously diagnosed with diabetes mellitus (DM) and hypertension, had morbid obesity, and had been diagnosed with chronic kidney disease (CKD) stage 3b. She had been treated for HIV, but she was not compliant with her therapy, and she also had a history of prior ovarian and vulvar malignancy, which were in remission at the time of presentation.

**Figure 1:**
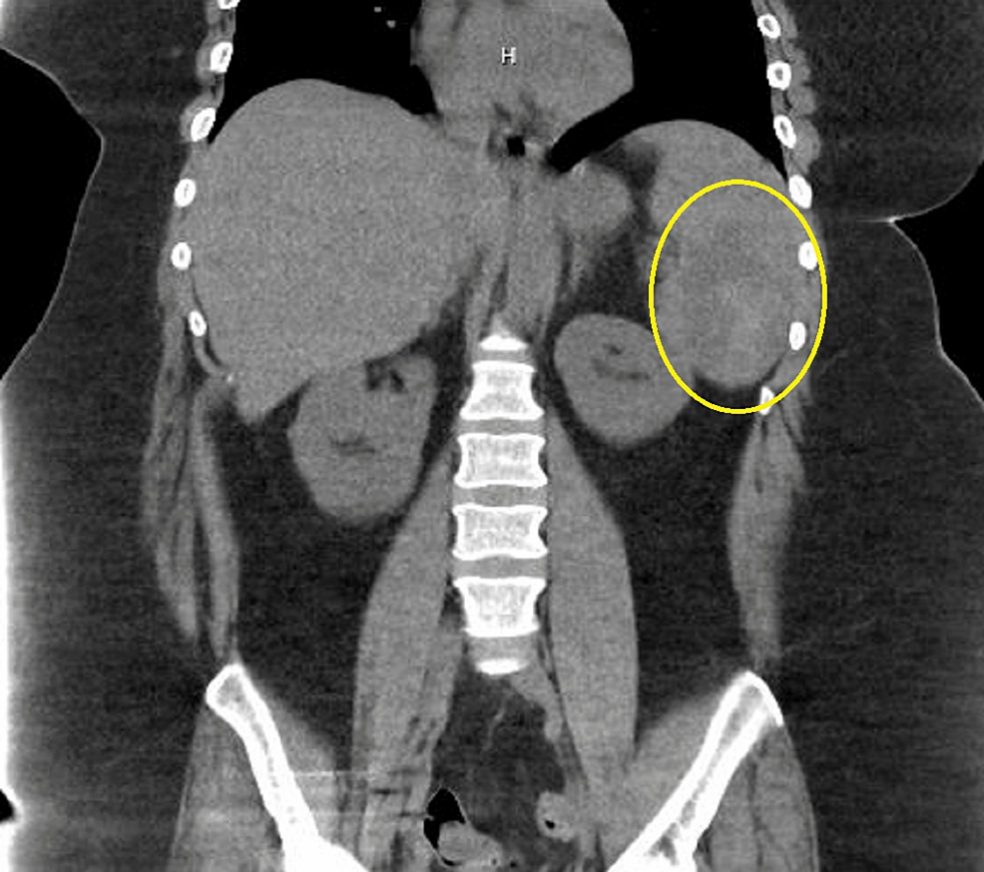
Coronal section of CT of the abdomen and pelvis showing an ill-defined lesion in the spleen (yellow circle) CT, computed tomography

Initial laboratory findings revealed that the patient had acquired immunodeficiency syndrome (AIDS) with an absolute CD4 count of 171 cells/mm3 and was in a hyperosmolar hyperglycemic state (HHS). Given her initial presentation, she was started on highly active antiretroviral therapy (HAART) and empiric antibiotic coverage with vancomycin and cefepime, dosed according to her present renal functions. Imaging on admission ruled out acute intracranial pathology and no concern for pneumonia; however, a computed tomography (CT) of the abdomen and pelvis showed an ill-defined lesion in the spleen (Figure 1).

**Figure 2:**
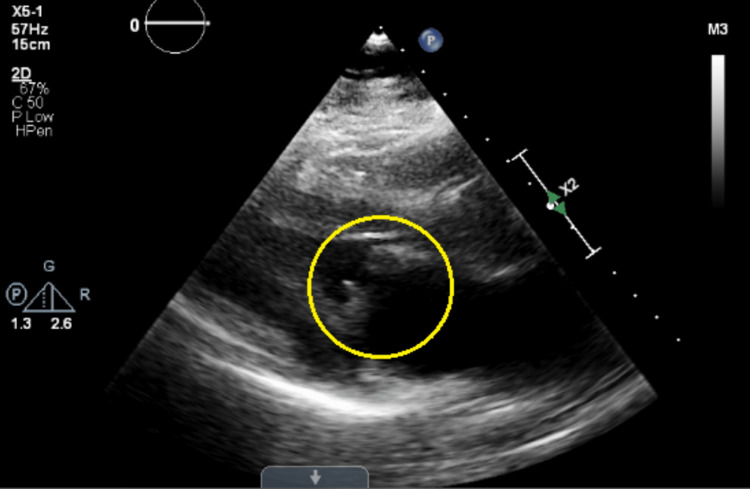
Transthoracic echocardiography showing thickening of mitral valve leaflets (yellow circle)

Blood cultures on the third day grew GBS, and antibiotics were narrowed down according to sensitivity to ceftriaxone. In addition, transthoracic echocardiography showed thickening of mitral valve leaflets with at least moderate valvular stenosis, further raising the suspicion of significant mitral valve disease and IE (Figure 2).

**Figure 3:**
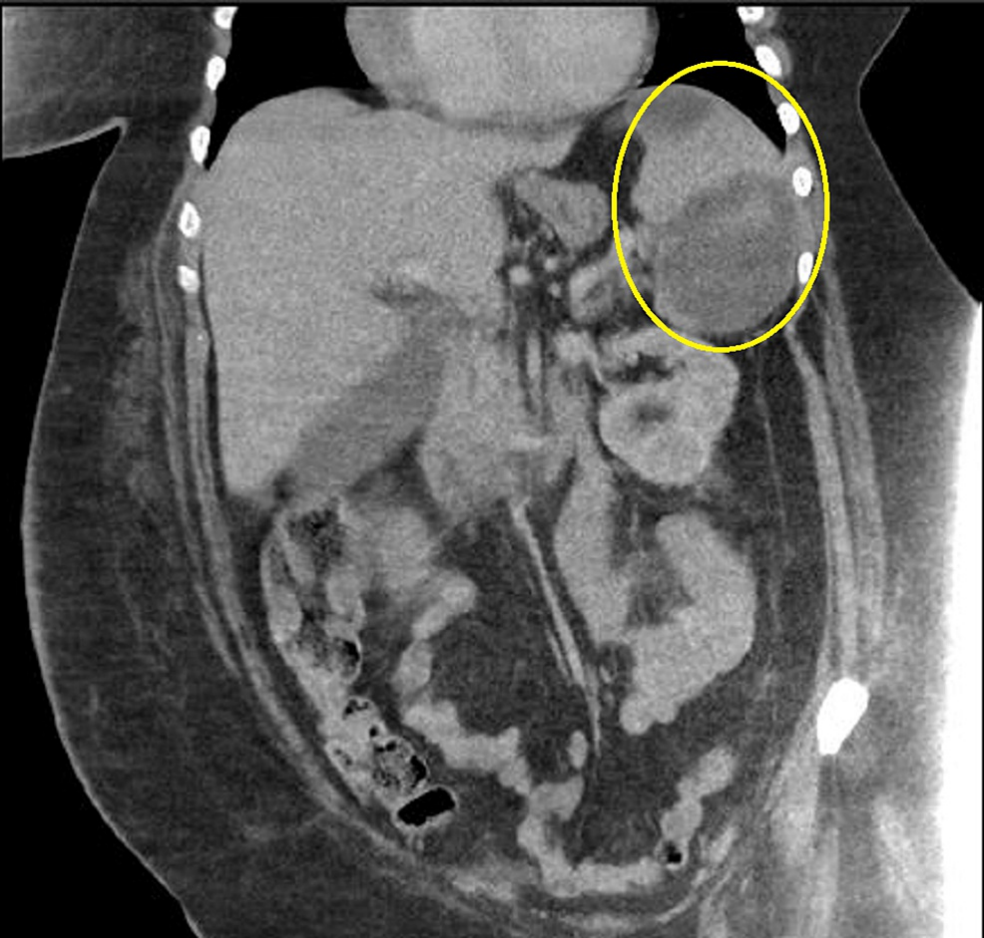
Coronal section of repeat CT scan of the abdomen showing the expansion of splenic lesion and multiple areas of decreased attenuation CT, computed tomography

**Figure 4:**
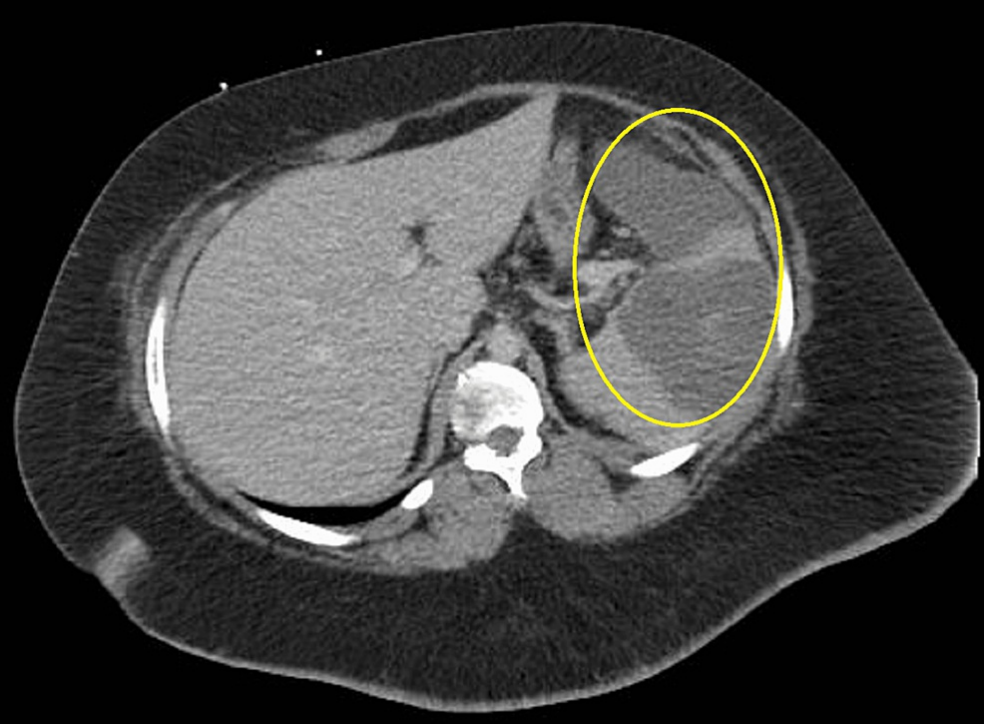
Axial section of CT scan of the abdomen showing the expansion of the lesion in the spleen with multiple areas of decreased attenuation CT, computed tomography

**Figure 5:**
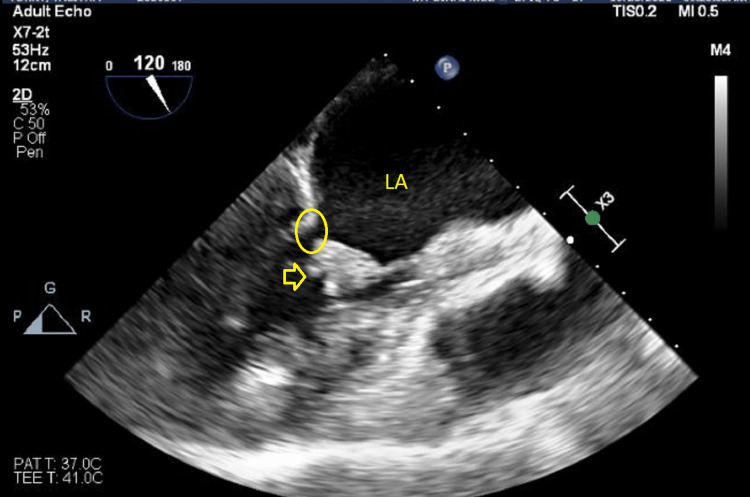
Transesophageal echocardiography showing infectious endocarditis with vegetations on the leaflets (yellow arrow) and severe mitral stenosis (yellow circle) in the setting of chronic rheumatic valve disease

The patient remained tachycardic and febrile despite proper antibiotic treatment and developed new-onset left lower back pain. A repeated CT scan of the abdomen showed an expansile process in the spleen with multiple areas of decreased attenuation compared to the first CT (Figures 3, 4). Later transesophageal echocardiography confirmed the suspicion of IE with vegetations on the anterior and posterior leaflets and severe mitral stenosis in the setting of chronic rheumatic valve disease (Figure 5).

Given the new findings, we attributed the splenic infarction to the confirmed diagnosis of IE of the mitral valve. The patient was evaluated for cardiothoracic surgery and deemed a poor candidate for surgery because of an operative risk of mortality of 69%, as per The Society of Thoracic Surgeons (STS) database scoring. Conservative management of the IE was recommended with prolonged antibiotic treatment with current ceftriaxone for six to eight weeks.

Her hospital stay was complicated with multiple conditions. She was noted to have purulent vaginal discharge, which was the potential source of her GBS infection, localized left T10 herpes zoster reactivation, intrasplenic hemorrhage causing anemia requiring blood transfusions, and pulmonary embolism. The patient was offered to have an embolization of the splenic artery with conservative management of her condition until further improvement or laparoscopic splenectomy pending clinical improvement; however, she requested a transfer for a second opinion to her primary center, where she was managed for her ovarian malignancy.

## Discussion

Despite the success of preventing neonatal GBS septicemia, the rate continues to increase in nonpregnant adults, with more than two-thirds of all GBS infections occurring in nonpregnant adults [6]. Most infections occur in patients over 60 years old and those with pre-existing conditions, including structural heart disease, chronic hemodialysis, and HIV [4]. DM is the most common comorbid condition occurring in 25% of patients with GBS endocarditis [4]. Our patient’s extensive history of hypertension, CKD, uncontrolled DM, and HIV with a low CD4 count puts her at high risk of suffering from GBS IE-related complications. IE of GBS is an uncommon finding in the setting of septicemia and is considered one of its most severe presentations associated with a high mortality rate [7,8]. Therefore, empiric treatment with a beta-lactam antibiotic and aminoglycoside is generally recommended as the treatment of choice for GBS bacteremia [8]. Considering that our patient has a history of CKD, we initially used vancomycin and cefepime as an alternative. The antibiotic therapy was later changed to ceftriaxone once the sensitivity cultures were available.

Splenic infarction is an unusual complication of IE [9]. However, it is a worrying sequela because it can lead to abscesses, pseudocysts, and hemorrhage [10]. A study by Lawrence et al. on 26 patients with splenic infarction showed that the most common clinical presentation of splenic infarction includes abdominal pain radiating to the left side (50%), elevated lactate dehydrogenase (80%), anemia, leukocytosis (56%), fever (36%), and splenomegaly [9,11].

Contrast-enhanced CT is the most sensitive imaging modality in diagnosing splenic infarction [10]. Once the splenic infarction has been discovered, it is paramount to investigate all possible etiologies to determine the source of the embolus. It is important to consider IE as a possible cause of splenic infarction, especially in a patient presenting with symptoms of acute systemic infection. Patients should be clinically screened for other signs and symptoms of IE. Early blood cultures on presentation and transthoracic echocardiogram soon after admission proved to be beneficial in diagnosing our patient.

First-line therapy for IE includes antibiotic treatment based on blood culture and sensitivity. Studies support valvular surgery as the treatment of choice for large thrombi and significant valvular involvement and splenectomy for large infarcts, recurrent abscesses, and expanding hematomas [10].

Our patient had vegetations on the anterior and posterior leaflets of the mitral valve, with the posterior vegetation being 1.1 × 1.0 cm and mobile; no perforations or abscesses were involved. The involvement of both mitral valve leaflets makes this a severe case of GBS IE. Our patient's risks of surgery were higher than the expected benefit for valvular surgery due to obesity, history of diabetes, anemia, immunocompromise, past medical history of cancer, the current presentation of active valvular endocarditis, and presentation of mitral stenosis. As per the STS database scoring, her operative risk of morbidity or mortality was 69% [12].

While surgery is the standard treatment for severe splenic infarct refractory to antibiotic treatment, endovascular embolization is a viable and safe alternative, especially in the case of a compromised cardiovascular system [13,14]. In addition, cases with high surgical risks, such as our patient, may benefit even more from this less invasive technique than a massive splenectomy before cardiac surgery [13,14]. Furthermore, there is evidence that splenic artery embolization improves the chances of splenic preservation, avoiding the need for a future splenectomy [14].

## Conclusions

*S. agalactiae*-associated IE is not a common entity; however, it could be a case in an individual with significant risk like the present case in an immunocompromised patient. Complications of IE need to be meticulously evaluated with a high degree of clinical suspicion with appropriate imaging in such cases. Additionally, nonsurgical treatment options for this subgroup of patients are vital due to the high risk of surgical morbidity and mortality.

## References

[1] Woods CJ (2023). Woods CJ: Group B Streptococcus (GBS) infections: practice essentials, background, pathophysiology. https://emedicine.medscape.com/article/229091-overview.

[2] Francois Watkins LK, McGee L, Schrag SJ (2019). Epidemiology of invasive group B streptococcal infections among nonpregnant adults in the United States, 2008-2016. JAMA Intern Med.

[3] D'Angelo M, Boretti I, Quattrocchi S (2019). Lethal infective endocarditis due to Streptococcus agalactiae in a man with a history of alcohol abuse: a case report. Medicine (Baltimore).

[4] Fujita H, Nakamura I, Tsukimori A, Sato A, Ohkusu K, Matsumoto T (2015). Severe infective endocarditis in a healthy adult due to Streptococcus agalactiae. Int J Infect Dis.

[5] Pringle SD, McCartney AC, Cobbe SM (1988). Spontaneous splenic rupture as complication of infective endocarditis. Int J Cardiol.

[6] Farley MM (2001). Group B streptococcal disease in nonpregnant adults. Clin Infect Dis.

[7] Sambola A, Miro JM, Tornos MP (2002). Streptococcus agalactiae infective endocarditis: analysis of 30 cases and review of the literature, 1962-1998. Clin Infect Dis.

[8] Baddour LM, Wilson WR, Bayer AS (2015). Infective endocarditis in adults: diagnosis, antimicrobial therapy, and management of complications: a scientific statement for healthcare professionals from the American Heart Association. Circulation.

[9] Lawrence YR, Pokroy R, Berlowitz D, Aharoni D, Hain D, Breuer GS (2010). Splenic infarction: an update on William Osler's observations. Isr Med Assoc J.

[10] Antopolsky M, Hiller N, Salameh S, Goldshtein B, Stalnikowicz R (2009). Splenic infarction: 10 years of experience. Am J Emerg Med.

[11] Wang CC, Lee CH, Chan CY, Chen HW (2009). Splenic infarction and abscess complicating infective endocarditis. Am J Emerg Med.

[12] (2023). Online STS risk calculator. https://riskcalc.sts.org/stswebriskcalc/calculate.

[13] Loffroy R, Guiu B, Cercueil JP (2008). Transcatheter arterial embolization of splenic artery aneurysms and pseudoaneurysms: short- and long-term results. Ann Vasc Surg.

[14] Shafi AM, Lee M, Balmforth D, Lall K (2020). Spontaneous splenic rupture following mitral valve replacement for infective endocarditis successfully managed with splenic artery embolisation. J Card Surg.

